# Rationales and functions of disliked music: An in-depth interview study

**DOI:** 10.1371/journal.pone.0263384

**Published:** 2022-02-15

**Authors:** Taren-Ida Ackermann, Julia Merrill

**Affiliations:** 1 Max Planck Institute for Empirical Aesthetics, Frankfurt am Main, Germany; 2 Institute of Music, University of Kassel, Kassel, Germany; University of Göttingen, GERMANY

## Abstract

**Background and objectives:**

With a few exceptions, musical taste has been researched via likes or preferences of certain types of music. The present study focuses on disliked music and takes a broad approach to cover explanatory strategies related to personal dislikes.

**Methods:**

In-depth interviews were conducted with 21 participants in five age groups. Interviewees were asked to prepare a list of their disliked music, and for each item they were asked about the reasons for the dislike. To ensure that the complexity and range of the participants’ dislikes and rationales were captured in the analysis, a structuring content analysis as a mostly theory-driven approach was combined with inductive category creation out of the interview data.

**Results:**

The most often mentioned type of dislike was musical style, followed by artist and genre. Five main reference points were identified for describing musical dislikes: the music itself, lyrics, performance, artist, and the people who listen to it. The identified rationales for disliked music were assigned to three larger categories: object-related reasons, such as music-compositional aspects, aesthetic dichotomies or lyrics; subject-related reasons, such as emotional or bodily effects, or discrepancies with the self-image; social reasons, which refer to one’s social environment and the taste judgments common to it (in-group) or to other groups of which the participants do not feel part of (out-group). Apart from the rationales for disliked music, the participants described specific reactions when they are confronted with their disliked music, such as emotional, physical, and social reactions.

**Conclusions:**

While musical dislikes have already been shown to fulfill important social functions, the current study extends the rationales to music-related and self-related reasons. Musical dislikes fulfill similar functions to liked music, such as preservation of a good mood, identity expression and construction, strengthening of group cohesion as well as social distinction.

## Introduction

With a few exceptions, musical taste has been researched via likes or preferences of certain types of music, painting an incomplete picture that cannot represent the evaluative diversity and complexity with which people approach, think and talk about their attitudes toward music. Understanding musical taste as an attitude, i.e., a general predisposition that is determined by a set of beliefs (opinions, positions, experiences) toward objects and leads to certain behavioral tendencies [1], an in-depth approach with qualitative measures seems promising in order to survey the attitudes toward music that play a role in the everyday lives of listeners. The goal of the present study is to investigate how people explain their negative musical judgments and thereby, extend musical taste and preference research by including negative attitudes into the concept of musical taste.

Given that attitudes are formed by beliefs, knowledge, and earlier experiences, it seems plausible to conceptualize negative attitudes toward music as a result of prior negative experiences with certain music, of knowledge and beliefs about it, and as a mismatch with acquired general beliefs about what is good and desirable in music, i.e., the individual music aesthetics. That dislikes must be acknowledged as a relevant part of musical taste can be learned from Bourdieus’s theory of social distinction [2]. Here, class-specific sets of liked and disliked music (and other cultural goods) are used as a resource for symbolic exclusion [3–5]. Further, the theory of omnivorousness today claims that higher social strata distinguish themselves from lower social strata by demonstrating broad cultural taste, i.e., by liking more (and disliking less) types of music [6–9].

While existing studies revealed which musical styles are often disliked and how they correlate with sociodemographic variables and, to a lesser degree, personality traits [10,11], individual rationales for disliking a certain type of music have not yet been studied comprehensively–very much in contrast to musical likes. Broadly, studies on music preference are based on two research approaches: On the one hand, researchers have been interested in which explanations are used by listeners to explain what they like, an approach that has been mainly pursued in qualitative studies [12–16]. On the other hand, the focus has been on the study of the functions music serves for an individual, where (preferred) music is evaluated by its suitability to certain purposes and on the use of music in everyday life [17–22]. While the first approach helps with answering the question of why participants like a particular music, a focus on functions and uses sheds light on the question of why participants generally like and listen to music.

Hence, the question arises as to which explanations are used for disliked music and whether functions other than social distinction exist. In some studies, the topic of dislikes came up with some of the participants (and were often reported parenthetically), pointing to certain similarities in explanations for musical likes as reported above. First, as a reason for disliked music (often either entire styles or specific songs), participants reported on music-related and performance-related aspects such as missing variation [14,23,24], repetitiveness, lack of complexity, incongruous or unexpected elements, missing (harmonic, melodic) resolution [24], and specific sound qualities (too loud, distorted, sound of instruments) [25]. Missing variation in the lyrics was also mentioned, i.e., meaningless or aggressive [14], overly simple (inane, stupid) and repetitive lyrics, overly sentimental or clichéd, misogynous sentiments and other biases, and contradictory or intelligible messages [24]. The voice of the singer was also subject to critique, mainly the singing characterization (annoying, yowling, monotonous, whiny), over-dramatic vocal effects [24], high-pitched voices, a nasal sound, or a death-growl [25,26]. The inability to sing along and missing variety between artists were also criticized [14].

Other reasons for dislikes pertained to perceived effects of the music on the listener and included unpleasant bodily feelings of disgust, nausea and pain, the perceived risk of physical harm, muscle tension, aggression, and the urge to escape [12,25]. “Inauthenticity” was also a reason for disliking music, as participants report on missing originality and honesty in the emotions and topics expressed in the music as well as copying other styles and artists in order to misuse this for their own success [12,16,23,27–30]. A supposedly strong focus on commercial success was also mentioned to foster a dislike [14,27], and a pretentious behavior by the artists (“wannabes and posers”; [24]). Aspects of production and certain forms of behavior and content (“sex and violence”) and supposedly negative effects of music on people/the youth and incompetence were criticized and disliked [14,27]. Further, painful and negative memories or experiences can lead to avoidance of certain music, and finally, the situation and context have an influence on the participants’ ability to bear their disliked music and determine how to react to it [24,25,31].

Based on these various and often single findings, a large range of reasons can be expected to account for the dislike of all sorts of music, such as peoples’ music aesthetics, earlier experiences with certain types of music, and general attitudes, views and beliefs. As the reported findings were rather a parenthetical product of studying musical preference, the current research took a qualitative approach with in-depth interviews to cover this expected variety.

### Degree and types of disliked music

Further findings suggest that there exist differences in the intensity of dislikes. Comments in online forums presented a range of judgments from mild rejections to vehement hatred and disgust of certain music [12], and people were reported strongly reacting to their musical dislikes and not simply giving neutral or ambivalent responses [32]. Another study found two “listener types,” one with a strong negative attitude and one with a rather neutral attitude toward the “aversive” music, showing that the first group experienced much stronger physically unpleasant feelings, a threat to their own musical identity and a violation of social and moral rules in response to aversive music than the other group [25].

Likewise musical likes can range from a mere “like it” to absolute love, one can expect that musical dislikes range from a mere “don’t like it” to hate. Therefore, the current study will investigate the reasons behind different degrees of disliked music.

Typically, studies on musical taste refer to music genres that are commonly used categories in everyday parlance and are thought to distinguish between types of music based on stylistic components, origins, aesthetics, meanings and represented social groups [33,34]. From a musicological point of view, one would rather call such types styles, whereas genre is typically defined as a type of composition that is characterized by its form, instrumentation, and function (e.g., Opera is a genre within the style of Classical music, whereas song is a genre that exists in almost any style). In the current study, the terms style and genre are used in their musicological sense, where participants reported a variety of musical dislikes, from general musical styles and genres to specific artists, albums, and individual songs.

### Functions of (disliked) music

The most common and clearly described function of positively valued music is its use in influencing one’s emotional state and mood [35,36]. Self or identity-related functions are also very important. In addition to identity construction, especially in adolescence [37–39], other functions include identity management, expression and reinforcement of individual values and attitudes, self-reflection and exploration, and trying out and expressing different aspects of personality [40–42] as well as finding meaning in life, or absorption and distraction from reality [43].

Functions of musical dislikes seem to be less straightforward identifiable, because typically, people do not listen to music they disapprove of. In communicating one’s attitudes toward music, however, such functions can be found in adolescents, where music is used to differentiate from other groups in order to discriminate the ‘out-group’ and at the same time to enhance the status of the ‘in-group’ and to increase their self-confidence [36]. Not only are the listeners of the other music discriminated against, also, the fans of the same (liked) music are valued higher than the fans of the disliked music [44,45]. Interestingly, within the in-group, it was still accepted if single members showed deviating attitudes and preferences, because the highest possible individuality and inadequateness within the group was valued [46].

One study explained the avoidance of aversive music with the process of emotional contagion [25]: The negative emotional and physical experience (through a mimicry of the music) might be interpreted as the function of disengaging with the music–which is then found aversive and disliked (note that cause and effect are hard to disentangle). These and other possible functions of musical dislikes need evaluation.

### Musical value judgments

It could be seen that sociological musical taste research included both liking and disliking of musical styles, since both are essential for social distinction and cohesion. However, there is also an individual, differentiated aesthetic judgment, which goes beyond a flat rejection of musical styles and the link with socioeconomic factors. Psychological studies of musical taste have investigated the functions and use of music and therefore, have been dominated by the study of musical preferences. This is where the current study comes in and explores the rationales for disliked music, which are the verbalized, individual reasons for disliking music. These rationales are based on an individual aesthetics, which can refer to different types of music and can include various reasons for disliking music.

Particularly relevant for the current study in this regard is a model on musical value judgements ([47,48]; further defined by [16]), which relates to the aforementioned attitudes toward music. Value judgments take place between three dimensions: The subject-dimension, i.e., the subjective level of judgment that includes personal liking, often based on prior experiences, and emotional and psychological functions, the object-dimension, that is judgments based on musical properties such as melody and rhythm, and the individually acquired idea of ‘good’ music, and the social-dimension, that is judgments in terms of social validity, which refers to the society as a whole but also to specific groups.

This model is of particular interest as it focuses on value judgements in which positive and negative judgements find their place, and as it does not already integrate the different reference points of rationales into the category system itself (such as particular musical styles or pieces). Therefore, it provides a frame for the present study on the psychological mechanisms of disliked music in which the participants’ explanations of their judgements were the focus.

### The present study

The present study focuses on disliked music and takes a broad approach to cover explanatory strategies related to personal dislikes. The study used in-depth interviews and followed research questions on (a) Which types of music are disliked?, (b) the explanatory strategies, i.e., how do the participants explain their musical dislikes?, (c) the impact of the participants’ musical dislikes, i.e. how do they react when confronted with this music or when they meet someone who likes this music?, (d) the extent to which the participants see a relationship between their musical dislikes and their self-image, (e) the strength of dislike, i.e., are there differences between the individual dislikes? Are some dislikes stronger than others?, (f) What functions do musical dislikes fulfill?

## Methods

### Ethics statement

All experimental procedures were approved by the Ethics Council of the Max Planck Society (No 2702–12), and were undertaken with written informed consent of each participant.

### Choice of research method

The basic principle of interviewing is a mindset of understanding participants as experts on their own experience and knowledge of the topic [49]. Therefore, in a semi-structured approach [50,51], the questions in the interview guide were used mostly as structuring elements in a flexible, advisory way rather than as binding wordings in order to react and communicate openly and flexibly with the interviewees [52]. Questions were constructed in a non-suggestive and neutral way that encouraged the participant to answer in full sentences.

Musical preferences change throughout one’s life and music, in general, does not always retain the same value in different phases of one’s life [35,53–55]. Therefore, care was taken to ensure that not only young adults and students were interviewed, but that a broad age spectrum was covered, using a stratified sampling approach with five different age groups. It is of note that no longitudinal effects can be covered with this cross-sectional approach.

### Participants

Participants were recruited via a pool of previous participants at the research institute, through the webpage of the institute, social networks (Facebook, Twitter), and flyers and posters in the city of Frankfurt am Main, Germany (libraries, community college).

Age groups consisted of four participants each with equal gender distribution and were grouped in blocks of ten years of age, starting at 18. The last group consisted of interviewees aged 58 and older. One test run was conducted, and because no changes had to be applied to the interview process, it was included in the main sample, adding to the number of participants (total *N* = 21, eleven female, mean age = 41.42, *SD* = 14.26, range 18–64 years). Therefore, the second age group consisted of five participants. Among the participants were eight students, five self-employed, five employees, a housewife, a doctoral student, and a pensioner. Nine of the participants had as highest educational attainment a High School diploma, twelve a university degree. Seven participants were professionally involved in music, 13 played an instrument.

### Procedure

After filling out the forms for data protection and giving consent about the recording of the interview, demographic data were collected (gender, age, education, profession, and musical activities) and participants were informed about the aim of the study and the procedure of the interview. The interviews took place in a room with a living room atmosphere. All interviews were conducted in German by the first author and all interviewees were native speakers.

Interviewees were asked beforehand to prepare a list of their disliked music. No instructions were given about how many items and what kind of music were to be prepared. For each item on the list, systematic questions were asked about the reasons for disliking this music, what the participant associates with this music, how they react when they come into contact with it, and what they think about the people who like it (see interview guideline in supplement). After all items had been reviewed, participants were asked to rate the strength of dislike of all their disliked music on an 11-point scale from neutral to most severe dislike. Afterwards, on a more general level, it was asked how the dislikes had developed and whether any changes had occurred, whether friends and social contacts shared the dislikes and to what extent the judgments of others influenced one’s judgment of music.

In a final phase, the interviewees were given the opportunity, still with ongoing audio recording, to make concluding comments and further remarks on the questions asked and topics discussed [52,56]. Interviews took about 50 to 100 minutes and monetary compensation was 15€.

### Qualitative content analysis

Qualitative content analysis is a method for analyzing qualitative data, mainly texts, in order to enable a structured description of the data. This is done by creating categories in a differentiated system and connecting them with passages in the text via codes. In doing so, the categories are firmly grounded in the data and therefore embedded in their context of communication [57–60]. To ensure that the complexity and range of the participants’ dislikes and rationales were captured in the analysis, two sub-methods of qualitative content analysis were combined: In essence, a structuring content analysis was used as a mostly theory-driven approach for the main categories and combined with inductive category creation out of the interview data.

### Transcription and coding

Transcription of the interviews was done manually and the coding was done with the software Atlas.ti, version 4.5. Transcription guidelines included accurate coding without any dialect, punctuation adjustment to written German, anonymization of all personal data, notation of breaks with duration, and notation of sounds like laughing or shouting in parentheses.

Before coding, all transcripts of the interviews were read through at least twice to make sure the content was well known to the coder. Based on the theory driven research questions and the resulting level of abstraction, categories were gradually developed inductively from the material. These categories were described using anchor examples and definitions and subsumed either under existing categories or included in the code system as a new category. The categories were revised at fixed intervals and summarized as needed. All decisions regarding the merging of categories, but also all considerations, questions, difficulties, and ambiguities regarding specific codes, were documented in the form of memos.

Data is provided as supporting information. It includes the codes and their translations from the interview study per participant and per category, Full quotes are published in the German dissertation by the first author [61].

## Results

### Descriptive analysis

#### Types of disliked music

**Fig 1:**
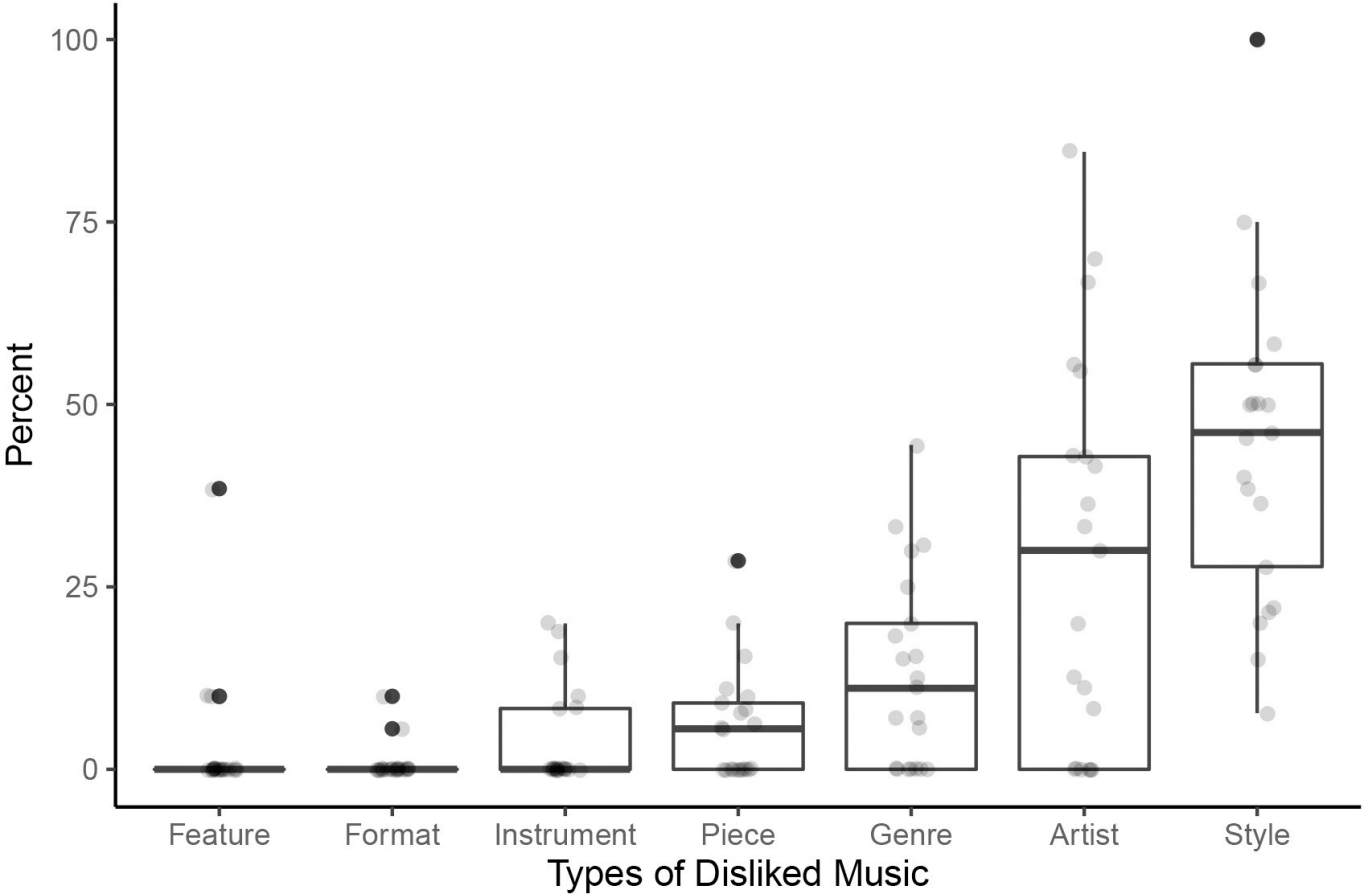
Percentages of types of disliked music. Boxplot showing the averaged frequencies in percent for each type of disliked music.

Using the prepared lists and verbal additions by the participants, a total number of 277 musical dislikes was counted, which ranged from 8 to 36 per participant with an average of 13 dislikes (see S1 Fig, supporting information). In order to account for this skewed distribution, the data was first averaged within participant and then across participants (see Fig 1, S1 Table, supporting information). On average, the most often mentioned type of dislike was musical style (44.4%), followed by artist (29.1%), genre (13.1%), specific pieces (6.1%), individual instruments (3.9%), musical characteristics (2.8%), and musical formats such as music-based TV shows (0.7%).

Schlager was the most frequently mentioned disliked style, while several substyles from Electronic Dance Music (EDM) were disliked such as Techno and House. The most disliked artists were from Rock music, and the most disliked genres belonged to Classical music (e.g., Opera). The majority of the single pieces listed belonged to Classical and Pop music (see S2 Table, supporting information).

#### Degree of dislike

**Fig 2:**
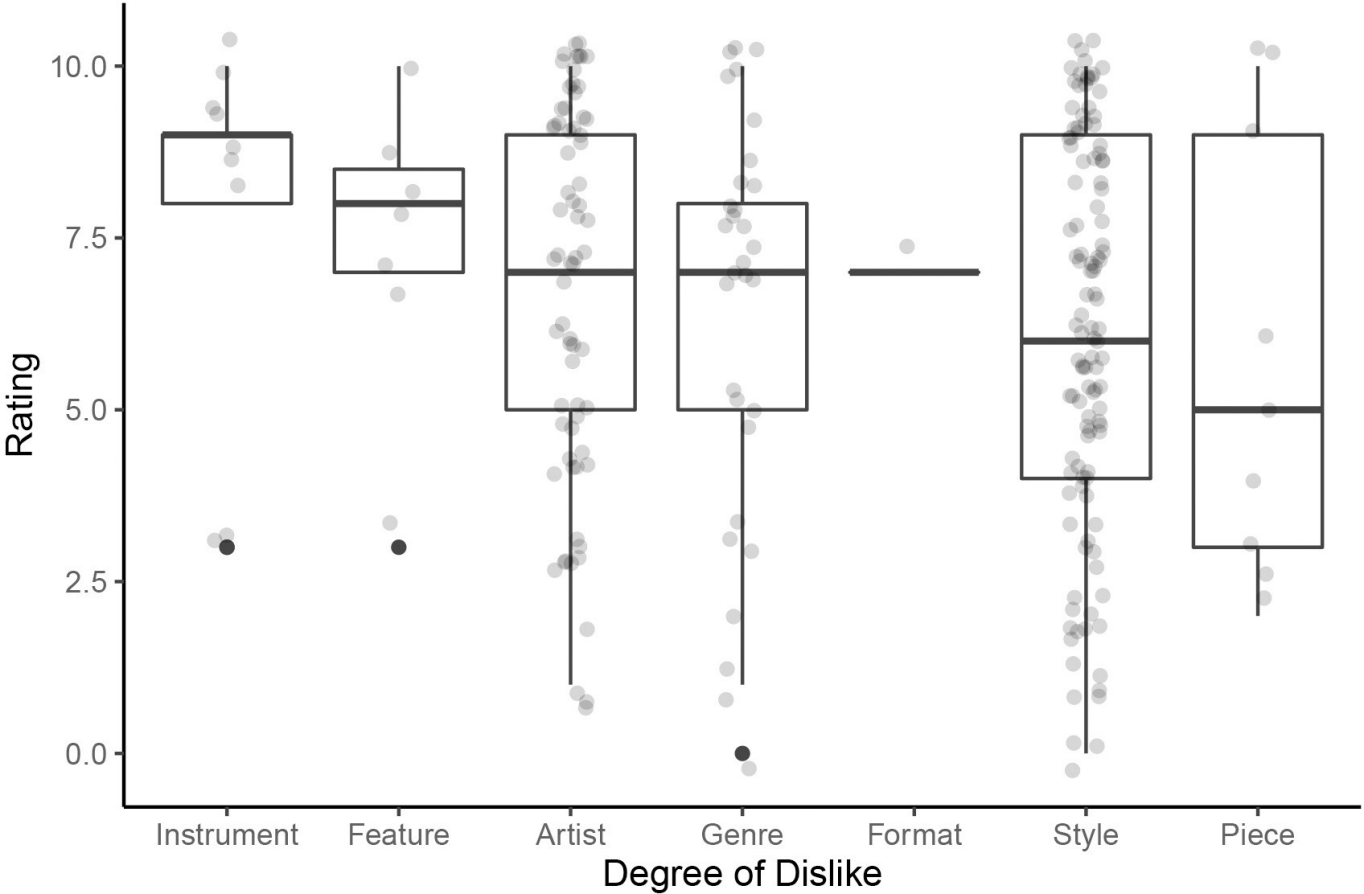
Dislike ratings per type. Participants rated their listed dislikes on an 11-point scale from 0 (neutral) to 10 (most severe dislike).

The dislikes on the lists were rated on an 11-point scale on the degree of dislike (*N* = 241), when applicable. Most frequently, the interviewees gave the most substantial dislike ratings (modal at 10 and 9 with 38 nominations each; see S2 Fig). The high degree of dislike (ratings ≥ 6) accounted for 63.1% of all dislikes evaluated. Even though the frequency distribution of the dislikes was thus clearly skewed, the participants used the range of the rating scale (see Fig 2 and S3 Table).

#### Reference points of dislikes

Before going into the rationales of disliked music itself, the actual reference points of the rationales had to be clarified, following the questions, a) to which aspects of the respective music does the dislike refer to?, and b) do the participants dislike the music as a whole, or can they name individual aspects on which their dislike is based?

Five main reference points were identified for describing musical dislikes during the interviews: the music itself, lyrics, performance, artist, and the people who listen to it. The majority (84.8%) of the rationales for disliked music were related to the music in combination with one or more of the other categories, and 40.4% of all dislikes were only justified because of the music and not because of other references to the lyrics or extra-musical categories. 32.9% of the rationales were related to the lyrics in combination with other categories, but only 2.2% solely to the lyrics. In the case of vocal music, music and lyrics cannot be separated, also because the voice of the artist does not only transfer content, but also sound qualities and emotions, and is therefore part of the musical expression. The artist is another reason for disliking music, which was the case in 31.4% in combination with other categories; in only one case the artist itself (the person) was the reason for the dislike. The participants referred to the listeners or fans of the disliked music in 20.2% of the rationales in combination with other reasons (in only 1.1% solely to the fans). The performance as a reason for a dislike (e.g., clothes, dance styles, TV formats, location, and setting) was used in 15.9% of the cases in combination and in only one case solely (see S4 and S5 Tables).

It is of note that these categories overlap and a clear separation is often impossible. Adding up the combinations of reference points without the music, the combinations amount to 12.3% (34 of 277), and adding up the combinations without the music *and* the lyrics, they amount to 5.8% (16 of 277). Hence, only a small percentage accounts for combinations between the artist, the listener/fan, and the performance. While 58.5% of the combinations include the music without the lyrics, only 6.5% include the lyrics without the music, and 26.4% include both. This shows that, typically, rationales for disliked music relate to a combination of reference points and only a few dislikes relate to only one particular point of reference.

Note that this was a purely descriptive analysis of the prevalence of reference points of rationales for disliked music. The reasons revealed in the following qualitative analysis relate to or coincide with these reference points, for example, the lyrics can be a reference point as well as a reason for the dislike itself. A person can dislike the lyrics because of the content or because of negative feelings evoked by it. It is also of note that this analysis does not show which reasons are more or less important or used more or less often.

### Qualitative analysis: Rationales for disliked music

The rationales for disliked music revealed by the qualitative analysis were assigned to three larger categories (Table 1).

**Table 1:** Rationales for disliked music.

Category	Subcategory
Object-related reasons	
composition	melody
	harmony
	rhythm
	structure
	loudness
performance	voice
	instruments
aesthetic dichotomies	variety vs. uniformity
	complexity vs. simplicity
	innovation vs. reproduction
lyrics	linguistic qualities
	content
kitsch*	
music vs. noise*	
Subject-related reasons	
emotions	perceived
	felt
	mood
bodily/physical	unpleasant
	discomfort
	volume
self-related	experiences
	exposure
normative	self-image
	beliefs
	values
	attitudes
authenticity and commerce*	
Social reasons	
in-group	family
	friends
out-group	the others
	stereotypes
	prejudice

* The categories mix reasons from several other categories.

#### Object-related reasons for disliked music

*Music-compositional aspects*. The participants referred to the melody, harmony, rhythm, structure/form, and loudness of the music. The disliked music was perceived as tuneless (“non-melodic”), “too melodic” and evaluated as “kitsch” (hokeyness) along with melodies being “not beautiful,” “too soft,” or “too smooth.” As melodic and harmonic features were often criticized together, music was likewise disliked if it was perceived as too “inharmonic,” “dissonant,” or “harmonically unfamiliar.” Concerning tempo and rhythm, participants criticized a too fast or slow tempo as well as too dominant, or not sufficiently pronounced, or monotonous rhythm. A varying rhythm as well as rhythm with a stimulating, thrilling effect, was described as something positive and desirable and if this was not given, the music was evaluated negatively.

Similarly, participants disliked music that did not have a clear or comprehensible structure, which was described as “abstract,” “drifting apart,” or as “noise.” In addition to a lack of structure, music was criticized when its form was perceived as “too simple,” “cheap,” or “predictable.” Some participants described a perceived loudness (not the actual adjustable volume) leading to unpleasant physical reactions.

Note, while in this analytical presentation following the content analysis these different ways of reasoning were presented separately, the participants mostly mixed and combined them to describe their dislikes. For example, a melody that is perceived as too simple usually goes hand in hand with a too simple and uniform rhythmic structure.

*Performance*. Other reasons for dislikes focused on performance aspects, such as the sound of specific instruments, which usually referred (rather broad and unspecific) to the overall sound of an instrument or piece as a whole, but also the singer’s voice, where the sound, the expression, and technique of the singer played a role (ranging from a “broken sound” to “shouting” or “shrieking”).

*Aesthetic dichotomies*. The explanations entailed general aesthetic categories, which can be applied to several aspects of the music and the lyrics. It needs to be considered that aesthetic criteria always depend on the discourse and are time-bound: While stylistic consistency is specifically valued in certain epochs (such as in the baroque) and within individual styles and associated subcultures (e.g., Country or Traditional German music, typically from the Alps region, and substyles of Electronic Dance Music), individuality and innovation are an essential part of the aesthetics and standards of other styles. Therefore, the aesthetic dichotomies now presented are used as an expression of the individual aesthetics of the participants and not as music-inherent qualities.

In the dichotomy variety and uniformity, the participants rated uniformity much more negatively than variety. Lack of variety or diversity was criticized in whole styles or genres as well as in individual musical elements such as rhythm or bass lines. Music was also disliked if the participants could not perceive significant differences between the different pieces of a style or an artist, of which “everything sounds the same.” This means that both uniformity within a piece as well as uniformity between pieces were disliked.

In the dichotomy of complexity and simplicity, the participants criticized when music or individual aspects of the music were too simple (with clearly derogatory terms such as “cheap,” “flat,” “primitive”). The level of differentiation of the statements was very different: While some respondents criticized the music of the disliked artist or composers as too simple, others referred to particular harmonic sequences (“tonic, dominant, subdominant”) or to a specific aspect such as the lyrics. Complexity in itself was not necessarily a positive characteristic of music for the participants. If the music becomes too sophisticated in its structure or sound, the listener may feel overwhelmed. It also seems to be essential to be able to “understand” the music. Behind this is the desire to be offered formal structures that are simple enough to remain catchy and understandable, and at the same time, sufficiently complex to provide inspiration and not become boring.

Related to innovation and reproduction, in most cases, experiments and new ideas were evaluated positively by the participants, and their absence was criticized. The participants argued for the most part with a mixture of musical similarity (“this is all the same”) and description of effect (“boring”). In some cases, however, they also criticized music covers and the use of existing melodic lines in other pieces in order to appeal to larger groups of listeners. Occasionally, participants also complained about the excessively experimental character of pieces.

The strongest form of musical dislike is possibly the denial of certain music actually being music (“this is not music”). By denying certain forms of music the status of music, participants deny not only its musical character but also any value that separates music as an art form and created work from mere noise (“for me, that is noise and not music”). This judgment was mentioned in the context of several other categories and is therefore categorized as cross-category.

*Lyrics*. Both the content of the lyrics as well as linguistic features were an issue to the participants. Disliked lyrics were perceived as too simple, too superficial, or unrealistic. In this kind of criticism, lyrics (particularly in Schlager and Traditional German music) were accused of taking up specific topics such as love, home, closeness to nature, or friendship, which were perceived as “clichéd” or “simple-minded,” as a representation of a “perfect world,” “ideal image” or “as in a fairy tale,” but not as a realistic representation of real relationships or honest feelings. Further aspects of the lyrics are discussed under “normative and self-related reasons.”

#### Subject-related reasons for disliked music

*Emotional effects*. A difference in explanations could be seen between the emotions perceived in the music and evoked by the music. The more different these two loci of emotions were, the more the participants themselves differentiated between the emotional expression of the music and their feelings. The most frequently mentioned perceived emotions in the disliked music were aggressiveness or anger, which were criticized mainly in the styles of Metal or Hip Hop, but also cheerfulness or good mood, which were most often mentioned in Schlager (in addition to not being honest or authentic).

In contrast to perceived emotions, the emotion felt was clearly in the negative valence range. Some participants reported that music can make them aggressive both by a match and a mismatch of both loci (e.g., the happy expression of Schlager making them angry). Others said that individual dislikes were caused by the music making them “sad,” “dragged down,” or “depressed.” These feelings shared a representation of emotional states that the interviewees perceive as negative and inescapable, and accordingly, dislike music that makes them feel this way. In addition to specific negative feelings, some participants also described a general decrease in their mood as an effect of their disliked music, which they said can sometimes last for some time.

Another often mentioned emotional reason for a dislike, however, was if the music “does not address” or “does not touch” them. In this case, respondents described disinterest or boredom as a reaction. Even if this lack of emotional effect did not induce a negative effect in the listener, it was evaluated negatively and led to a dislike of the respective music.

*Bodily/physical effects*. In addition to emotional effects, participants reported unpleasant physical reactions to music. These descriptions remained rather general and imprecise: the music was “unpleasant,” “exhausting,” or “does not do one well.” In most cases, the participants used it to describe a state of general tension or exertion that they find genuinely unpleasant. In a sense, music acts against their will on them and their bodies (“harassment”) without them being able to escape it. For some participants, this feeling of physical discomfort is related to the volume of the music, for others the overall sound. In addition to general discomfort, however, individual participants also report specific physical symptoms in response to the disliked music such as headaches or pain in the ears, but also nausea, a racing heart, uneasiness, and intense tension.

In addition to the negative effects of music described above, participants criticized a discrepancy between desired and actual effects. This is primarily about a lack of physical activation such as lack of “danceability” or other physical stimulation.

*Self-related and normative reasons*. The reasons in this category can be roughly divided into two subcategories: First, participants referred to certain, mostly negative, experiences in their past, and an excessive exposure of previously positively or neutrally assessed music (which was “heard too often”) that led to the dislike (self-related reasons). Second, they justified their judgments based on their self-image and related attitudes, which conflicted with attitudes associated with the disliked music (political, religious, or moral attitudes; normative reasons). Some participants described a rather broad discrepancy between their self-image and the qualities attributed to music, without going into the details of which qualities of music do not “fit.” Others, however, stated in more detail which aspects of the music were incongruent with their own opinions or values (based on song lyrics or other signs), mainly referring to artists who are clearly politically classifiable.

*Authenticity and commerce*. Participants also evaluated music by confirming or denying authenticity. As this category mixes reasons from several other categories, it is categorized as cross-category. Music is “good” if it is “an expression of personal things” and transports “honest” feelings and does not serve the “interests of the music industry.” Hence, a lack of authenticity was closely linked to the aspects of mainstream and commerce. Behind the criticism of certain music taken as “inauthentic” was, in most cases, the claim that music as an art form should be independent of economic success and trends. Compromises by artists who are guided by market interests were therefore disliked, as are deals with overpriced concert tickets and merchandise. At the same time, the artists’ attitude towards their music and the market itself was also evaluated, as they are required to perform out of belief and identify with their music instead of routinely “doing their job.”

The attribution “not authentic” was also used in an emotional context. Participants expected that the emotions presented in the music either describe the emotional life of the artists themselves or are a realistic reflection of emotional states that they know and can comprehend. If neither one nor the other is given, the interviewees perceived the emotional content of the music as “wrong” and “exaggerated.” Some participants emphasized the subjective character of this criticism: they individually do not feel addressed by the music, or rate the music as not honest or exaggerated. This means that the focus is less on distancing oneself from other listeners than on the need to identify with the content and emotional expression of the music or the artists and their environment, or at least to agree with them in their requirements of art and music. Accordingly, music that does not meet these requirements or does not offer sufficient potential for identification and agreement is regarded as inauthentic.

#### Social reasons for disliked music

The social reasons could be divided into arguments that refer either to one’s social environment and the taste judgments common to it (in-group) or to other groups of which the participants do not feel part of (out-group). These justifications were often connected with prejudices and stereotypes about the corresponding group. In-group and out-group reasons were also not mutually exclusive and were sometimes used to complement one another.

Membership of a particular social group, be it family, friends, or the general social environment, determined only a relatively small part of the reasons for dislike. Defying a code of attitude and judgement about the common dislikes in the group would potentially lead to consequences for the individual in that their reputation and standing would suffer (“one is not allowed to find it good,” “that is simply uncool, so to speak”). The disliked music can also refer to a certain level of education within the in-group (“no one who has any sense or taste at all listens to such things”).

Stereotypical attributions played an essential role in the distinction between the listeners, fan groups, or artists of the disliked music and the outside world. Of great importance here again was the political attitude of the musicians and fans, in addition to the associated educational level and the attributed character traits, which were, in most cases, more or less explicitly formulated stereotypes. Participants also evaluated the bands and singers in terms of how likable they appear and how they present themselves in interviews or at concerts. Disliked styles that were closer to certain subcultures than others offered additional starting points for stereotyping. A good example is Heavy Metal, whose fans express their musical preference in the form of clothing (“band T-shirts and dark colors”) and appearance (“long hair, beards”). In the interviews, several participants expressed prejudices against the Heavy Metal listeners and musicians and often had negative associations.

In addition to certain, mostly negative, character traits, the interviewees also attributed psychological abnormalities or disorders to the fans or associated them with substance and alcohol abuse or criminal behavior. However, in most cases, it was more about sympathy and the general impression that the artists made on the interviewees. These impressions were generated by the pseudo-knowledge the participants had mostly gained through social media.

### Reactions to disliked music and its fans

Apart from the rationales for disliked music, the participants described specific reactions when they are confronted with their disliked music: On the one hand, they reported (1) emotional and (2) physical reactions to the confrontation with the disliked music, which were already presented as an unpleasant effect of the music. On the other hand, they reported (3) action implications and (4) social reactions in the case that they met someone who appreciated the disliked music.

While some dislikes could be tolerated by the participants for a certain time, others led to clearly described avoidance behavior. If certain disliked music was played, participants either changed the music or switched it off or even left the room. With stronger dislikes, they also avoided places where they would be confronted with the music such as certain clubs, discos, and parties. It was unanimously described that the social environment can influence one’s behavior toward disliked music: Accompanied by friends, several participants reported that disliked music was more tolerable. In most cases, participants said that someone else’s preference of their disliked music would have no effect on them, or that they would even respond positively. Only in the case of differences in (political or religious) attitudes they would also avoid the supporters of their disliked music and, if necessary, break off a conversation if their counterpart was to identify themselves as a fan.

## Discussion

The results show a wide variety of rationales for disliked music and extend previous findings from musical taste research. Participants report on various musical dislikes to different degrees using explanations that represent three major categories of musical value judgments, i.e., the subject, the object, and the social (applied post-hoc based on [47]). Broadly, music is disliked if it does not comply to ones beliefs, the self-image, or the individual aesthetics, i.e., what one expects from the music. Thus, this study succeeded in showing that musical dislikes are a complex, multidimensional component of musical taste and not just the negative antithesis of musical preferences. The current findings provide further confirmation that surveys of musical taste working exclusively with musical style categories omit some of the types of music used by listeners and thus a substantial part of the participants’ musical tastes.

### Rationales for disliked music

Although disliked styles form the largest share among the listed “musical dislikes,” the participants also report on artists, pieces, existing as well as made-up genres, certain instruments and musical characteristics. Further, the analysis of the reference points of the rationales (i.e., what a specific reason is actually referring to), showed that a large part of the reasons for disliked music relate to the music itself (in about 40% of the cases) and to the music in combination with other reference points such as the lyrics, the artist, and the fans/listeners of the music (about 85%).

The rationales comprise reasons that describe the object (e.g., musical features or aesthetic dichotomies), the subject, and/or the social environment. With regard to subject-related or self-related reasons, music is disliked if the associated content, characteristics, or behaviors do not match those of the listener (cf. [1]), which is expressed by the participants, who see correspondences between their self-image and their negative attitudes toward certain music. Social reasons in the present study focus on the participants’ reasons specifically related to other people and the social environment. Reasons related to the out-group, fed by stereotypes and prejudices about the fans and listeners of the disliked music, were more frequent than reasons related to the in-group. The dislike of certain types of music served in this context to strengthen the inner cohesion of the group by confirming the existence of clearly defined antagonists and associated values which one shares and may defend externally.

It is of note that the categories presented show overlap, and the participants combine different categories for the dislikes. In some cases, it is also difficult to distinguish purely negative descriptions of the music from reasons for the dislikes. In addition to the three larger categories, three cross-categories can be found in the explanations of the participants, which contain aspects of all larger categories, that is authenticity in combination with marketing strategies, and the reference to certain music as kitsch and as noise or “non-music”.

### Missing an ideal mean of music: Too much or too little

In many cases, the respondents justified their dislikes by using deviations from an ideal expression of certain characteristics, that is “too much” or “too little.” A similar observation was made in another study, which found judgments of various likes and dislikes referring positively to the “correct” extent of a characteristic [62]. This insight is used in consumer research in the form of the “just-about-right scale” (JAR-scale; [63]), which evaluates the properties of an object with an ideal center (“just about right”) between the endpoints “too much / too strong” and “too little / too weak,” which has been used in music-related studies evaluating piano [64] or vocal performances [65]. However, it does not seem that both deviations are equally relevant for all characteristics of disliked music. Participants also reported that certain negative deviations can, to a certain extent, be compensated for by other positive characteristics.

This interrelation of a “too much” and “too little” is reminiscent of Berlyne’s idea of the inverted U-shape to explain preference occurring with arousal potential. Here, collative variables such as familiarity and complexity are usually taken into account [66], but the current results point to the idea that listeners expect an optimal experience which equates to being optimally aroused and engaged by many different variables [67,68]. This is at least the ideal they seem to be striving for in order to like the music. One variable directly points to the findings around the inverted U-shape, that is, participants report a dislike because they “have heard it too often”, which leads to displeasure due to over-exposure.

### Functions of musical dislikes

For the musical preferences of Western listeners, several studies have shown that positive judgments of certain music are closely linked to its fulfillment of certain functions [69–71]. Especially the possibility to express one’s own identity by means of music, but also the use of music to establish contact with others, its ability to trigger strong positive emotions, or to express one’s own values in it, determine the strength of the preferences [22].

Notably, the current participants hardly ever directly use functions to describe their dislikes. Hence, the functions of musical dislikes have to be inferred from the participants’ explanations and mainly refer to the functions of communicating the dislikes and not the use of the music itself (i.e., listening to it).

First, as with liked music, identity expression plays a major role. Many participants drew direct parallels between their self-image and the characteristics and attitudes in the music, which they describe as not fitting or belonging to them. Hence, musical dislikes serve the self-presentation in social contexts, but are equally relevant as a psychological process for strengthening one’s own self-image and self-esteem by distancing oneself from the negative self [72].

Two related functions of musical dislikes represent the avoidance of negative emotional and physical states as well as negative or painful memories triggered by the music. Hence, while preferred music is chosen to enhance the mood [43], or trigger mind wandering and reminiscence [17], disliked music is avoided in order to avoid unpleasant feelings. It is of note that music can also be disliked if it does not fulfill the expected functions of preferred music.

The most indirect function of musical dislikes might be the demonstration of musical competence: Calling music “bad” or denying it the status of actually being music or by calling it noise as well as criticizing certain musical aspects, serves emphasizing one’s own knowledge and “good taste.” By communicating one’s own musical standard and requirements of “good music,” the participants communicate their musical education, and the extent of their musical taste and by this, inform about the cultural capital they possess [2,12,13]. The expression of one’s own musical competence is thus closely linked to the function of creating and strengthening social cohesion and distinction.

Criticism related to authenticity and commerciality is also related to social distinction. If the liked music becomes too successful, it loses its function as a distinguishing feature that creates (group) identity and is rejected. In addition, the dislike of music with contradicting political, moral, or religious stands also serves to position oneself in society (e.g., as anti-fascist or left-wing). Rejecting certain music for these reasons means likewise avoiding places and events with this music, and therefore the risk of socially engaging with people who identify with these opinions.

### Types and degree of disliked music

Foremost, participants reported on the dislikes referring to musical styles (44%) and artists (30%). While on the level of styles, primarily Schlager, Traditional German music and Metal were disliked, the substyles were mainly from Electronic Dance Music, non-European music, Metal and Rock. The disliked artists were predominantly from Rock, Pop, Schlager and Classical music. Three observations can be made from this: First, Schlager, Traditional German music and Metal were also among the most disliked styles in other studies (e.g., Schlager and Traditional German music in Germany; [73], and Metal in the US; [3]; for more recent dynamics of dislikes among different age groups, see [4]). Second, naming certain artists needs a certain level of familiarity with the style, which is the case for Pop, Schlager and Classical music to an extent, which make use of the celebrity focus in the media. Notably, people can dislike an artist or a genre within a generally liked musical style, which has consequences for future musical taste studies. Third, the findings show how important it is to not only focus on style evaluations but also on genre evaluations within a style. In Classical music, for example, dislikes of the overall style seem to be rare [3,32,74], but disliking individual artists (e.g., a famous singer) and genres (e.g., Opera) do play a role (underlined by the number of disliked genres in Classical music in the current study).

With regard to the degree of dislike, reviewing the quantitative evaluation on how much participants disliked their mentioned music, about one third of the dislikes was at and below the midpoint of the rating scale, showing that music exists that is disliked to a lesser degree. Whether different rationales or certain individual variables such as personality or musical sophistication apply differently to strong and slight dislikes will need to be investigated in future studies.

### Limitations

The sample was controlled for by age and gender but not for any other demographic or socioeconomic variable. This resulted in a higher educated sample with a higher interest in music, i.e., a third of the participants were professionally involved with music (practitioners, scientists, journalists). This observation underlines a common relation between interest in music and interest in music-related studies. This relationship seems to be even stronger when invited to an interview study where participants are required to talk about music. Besides the obvious limitations, the advantage of this higher educated sample was that they were all able to articulate the reasons for their disliked music in much detail.

Further, the higher education level might have led participants to dislike certain musical styles more often than others. For example, as some sociological studies on musical taste have shown, the preference of traditional music (and its variations) is linked to a lower socioeconomic status. In the current study, German Schlager was often disliked. Still, the range of musical styles included in this study was very wide, but certain reasons for musical dislikes might apply to certain styles (or single artists or songs) only. How they do exactly, needs further investigation.

## Conclusion

Conceptualizing musical dislikes being an inherent part of the concept of musical taste and therefore an inherent part of music-related behavior and its consequences for everyday life, this study showed the range and extent of the rationales underlying disliked music. While musical dislikes have already been shown to fulfill important social functions, the current study extends the rationales to music-related and self-related reasons.

Musical dislikes fulfill similar functions to liked music, such as preservation of a good mood, identity expression and construction, strengthening of group cohesion as well as social distinction. However, in contrast to the reasons behind liking, the justifications are mainly related to very specific musical characteristics, the content, evoked feelings (or lack of), and aesthetic expectations of the music.

The current study showed how many different aesthetic criteria underlie everyday evaluations of music. Hence, focusing musical taste research exclusively on listeners’ musical preferences cannot account for this diversity.

## Supporting information

S1 Fig Frequencies of types of disliked music per participant. (PDF)

S2 Fig Histogram of the degree of dislike. (PDF)

S1 Table Mean and standard deviation of the percentages of types of disliked music. (PDF)

S2 Table Frequencies of disliked musical styles per type. <**/SI_Caption>**
(PDF)

S3 Table Mean dislike rating per musical style and type. (PDF)

S4 Table Frequencies of reference points of disliked music and their combinations. (PDF)

S5 Table Sum of frequencies of reference points of disliked music. (PDF)

S1 File (PDF)

S1 Data (XLSX)
