# Supplementary figures and images for: Rationales and functions of disliked music: An in-depth interview study
Source: PLoS One. 2022 Feb 15;17(2):e0263384. doi: 10.1371/journal.pone.0263384 (PMC8846515; doi:10.1371/journal.pone.0263384)

**Fig S1**

*Frequencies of Types of Disliked Music per Participant*

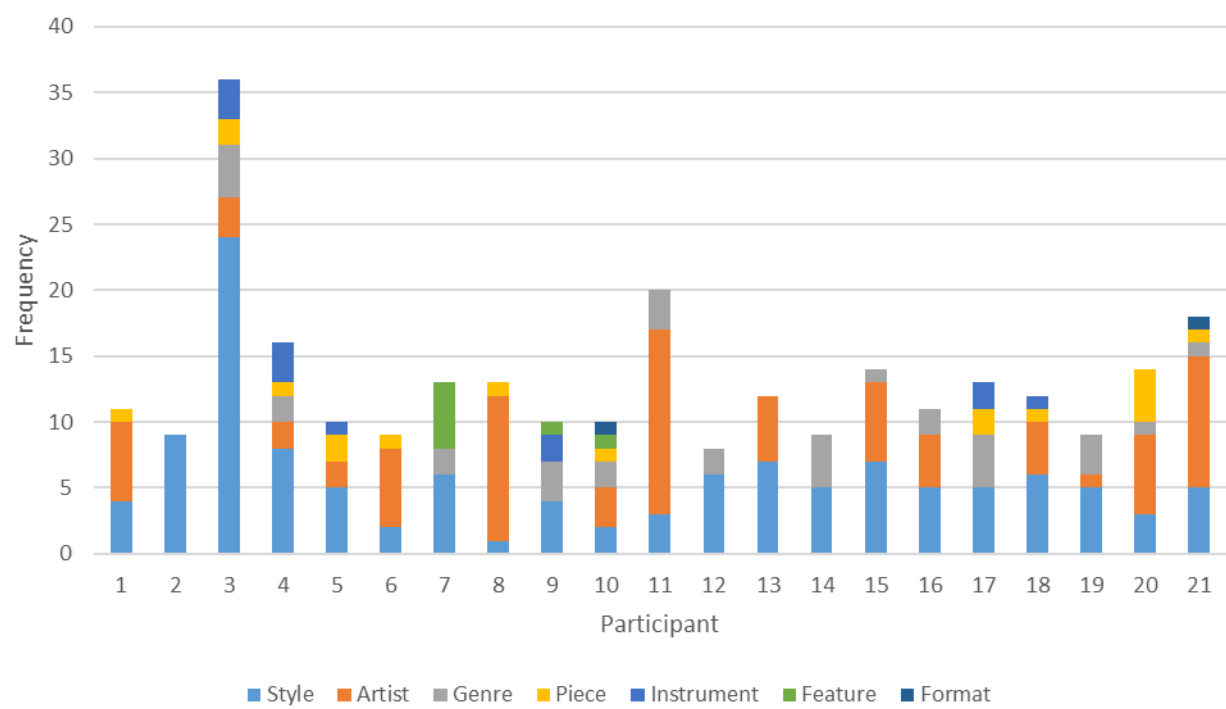

Supplement: S1 Fig — (PDF) [file pone.0263384.s001.pdf]
