# Supplementary material for: Rationales and functions of disliked music: An in-depth interview study
Source: PLoS One. 2022 Feb 15;17(2):e0263384. doi: 10.1371/journal.pone.0263384 (PMC8846515; doi:10.1371/journal.pone.0263384)
Supplement: S2 Fig — (PDF) [file pone.0263384.s002.pdf]

Fig S2

Histogram of the Degree of Dislike

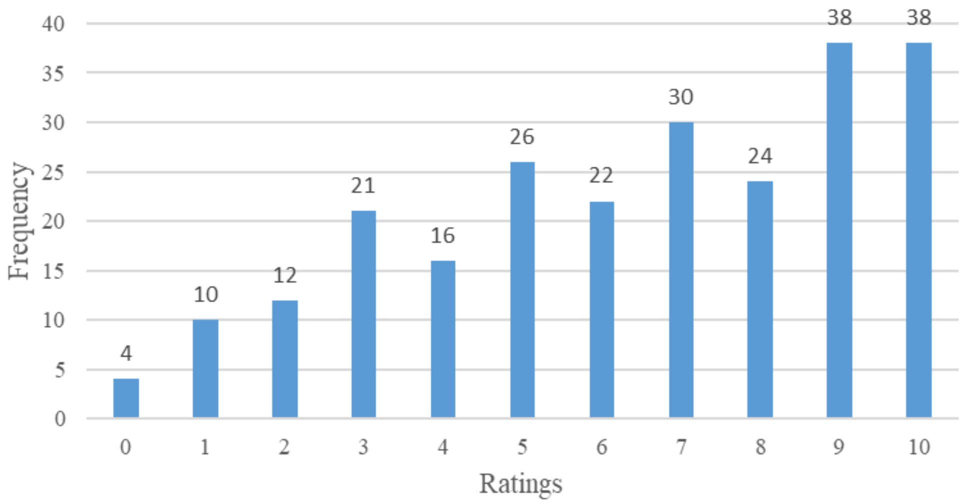

Note. Ratings of degree of dislike (neutral to most severe dislike) of the rated dislikes (N = 241).
