# Supplementary material for: Rationales and functions of disliked music: An in-depth interview study
Source: PLoS One. 2022 Feb 15;17(2):e0263384. doi: 10.1371/journal.pone.0263384 (PMC8846515; doi:10.1371/journal.pone.0263384)
Supplement: S1 Table — (PDF) [file pone.0263384.s003.pdf]

Table S1

Mean and Standard Deviation of the Percentages of Types of Disliked Music

	Style	Artist	Genre	Piece	Instrument	Feature	Format
***M* %**	44.37	29.07	13.12	6.07	3.85	2.78	0.74
***SD* %**	21.06	26.11	13.00	7.60	6.61	8.50	2.38
