# Supplementary material for: Rationales and functions of disliked music: An in-depth interview study
Source: PLoS One. 2022 Feb 15;17(2):e0263384. doi: 10.1371/journal.pone.0263384 (PMC8846515; doi:10.1371/journal.pone.0263384)
Supplement: S2 Table — (PDF) [file pone.0263384.s004.pdf]

Table S2

Frequencies of Disliked Musical Styles per Type

Style Categories	Meta-style	Sub-style	Artist	Genre	Piece	Total
**Rock**	1	9	27	1	2	40
**EDM**	2	21	5	4	0	32
**Pop**	1	5	14	7	5	32
**Schlager**	13	1	14	1	2	31
**Classical music**	1	0	10	12	6	29
**Non-European music**	0	19	0	4	0	23
**Heavy Metal**	7	11	4	0	0	22
**Traditional German music**	8	2	1	1	0	12
**Hip Hop / Rap**	4	2	4	0	0	10
**Jazz**	3	4	0	0	0	7
**Country**	4	0	1	0	1	6
**Reggae**	2	0	0	0	1	3
**Soul**	1	0	1	1	0	3
**Singer**	0	1	2	0	0	3
**Blues**	0	0	0	1	0	1
**Total**	47	75	83	32	17	254

Note. To assign the disliked artists to the meta- and substyles, the most frequent tags from the online music service *last.fm* were used.
