# Supplementary material for: Rationales and functions of disliked music: An in-depth interview study
Source: PLoS One. 2022 Feb 15;17(2):e0263384. doi: 10.1371/journal.pone.0263384 (PMC8846515; doi:10.1371/journal.pone.0263384)
Supplement: S3 Table — (PDF) [file pone.0263384.s005.pdf]

Table S3

Mean Dislike Rating per Musical Style and Type

	Artist	Genre	Piece	Style	Mean
**Non-European music**		8,75		6,47	6,90
**Blues**		10,00			10,00
**Country**			9,00	5,00	5,80
**EDM**	7,00	4,00		6,26	6,16
**Hip Hop**	5,50			6,40	6,00
**Jazz**				7,50	7,50
**Classical music**	3,57	6,73	6,00	9,00	5,70
**Heavy Metal**	6,67			7,71	7,55
**Pop**	6,82	5,17	4,40	3,17	5,25
**Reggae**				3,00	3,00
**Rock**	7,75	10,00	5,00	6,33	7,37
**Schlager**	5,73	7,00	10,00	5,29	5,70
**Singer- Songwriter**	10,00			5,00	8,33
**Soul**	10,00	0,00		1,00	3,67
**Traditional German music**	9,00	9,00		5,44	6,09
**Mean**	6,74	6,48	5,78	6,10	6,34
