# Supplementary material for: Rationales and functions of disliked music: An in-depth interview study
Source: PLoS One. 2022 Feb 15;17(2):e0263384. doi: 10.1371/journal.pone.0263384 (PMC8846515; doi:10.1371/journal.pone.0263384)
Supplement: S4 Table — (PDF) [file pone.0263384.s006.pdf]

Table S4

Frequencies of Reference Points of Disliked Music and Their Combinations

**Total**	**277**		**Style**	**122**		**Artist**	**83**		**Genre**	**34**	
Music:	112	40,4%	Music:	55	44,7%	Music:	12	14,5%	Music:	18	56,3%
Lyrics:	6	2,2%	Lyrics:	3	2,4%	Lyrics:	1	1,2%	Lyrics:	1	3,1%
Artist:	1	0,4%	Artist:	0		Artist:	1	1,2%	Artist:	0	
Listener (F):	3	1,1%	Listener (F):	2	1,6%	Listener (F):	1	1,2%	Listener (F):	0	
Performance:	1	0,4%	Performance:	1	0,8%	Performance:	0	0,0%	Performance:	0	
M L	27	9,8%	M L	12	9,8%	M L	5	6,0%	M L	7	21,9%
M A	17	6,1%	M A	0		M A	15	18,1%	M A	1	3,1%
M F	7	2,5%	M F	5	4,1%	M F	0	0,0%	M F	2	6,3%
M P	9	3,3%	M P	5	4,1%	M P	0	0,0%	M P	2	6,3%
L A	7	2,5%	L A	1	0,8%	L A	6	7,2%	L A	0	
L F	0		L F	0		L F	0		L F	0	
L P	0		L P	0		L P	0		L P	0	
A F	4	1,4%	A F	0		A F	4	4,8%	A F	0	
A P	2	0,7%	A P	0		A P	2	2,4%	A P	0	
F P	2	0,7%	F P	0		F P	0		F P	0	
M L A	17	6,1%	M L A	5	4,1%	M L A	11	13,3%	M L A	1	3,1%
M L F	5	1,8%	M L F	5	4,1%	M L F	0		M L F	0	
M L P	3	1,1%	M L P	1	0,8%	M L P	0		M L P	1	3,1%
M A F	5	1,8%	M A F	3	2,4%	M A F	2	2,4%	M A F	0	
M A P	5	1,8%	M A P	2	1,6%	M A P	3	3,6%	M A P	0	
M F P	5	1,8%	M F P	3	2,4%	M F P	0		M F P	1	3,1%
L A F	3	1,1%	L A F	1	0,8%	L A F	2	2,4%	L A F	0	
L A P	0		L A P	0		L A P	0		L A P	0	
L F P	0		L F P	0		L F P	0		L F P	0	
A F P	3	1,1%	A F P	1	0,8%	A F P	2	2,4%	A F P	0	
M L A F	11	4,0%	M L A F	3	2,4%	M L A F	8	9,6%	M L A F	0	
M L A P	6	2,2%	M L A P	3	2,4%	M L A P	3	3,6%	M L A P	0	
M L H P	2	0,7%	M L H P	2	1,6%	M L H P	0		M L H P	0	
M A F P	2	0,7%	M A F P	2	1,6%	M A F P	0		M A F P	0	
L A F P	2	0,7%	L A F P	1	0,8%	L A F P	1	1,2%	L A F P	0	
M L A F P	2	0,7%	M L A F P	2	1,6%	M L A F P	0		M L A F P	0	
not specified	8	2,9%	not specified	4	3,3%	not specified	4	4,8%	not specified	0	

Note. M = Music, L = Lyrics, A = Artist, F = Fan/Listener, P = Performance
