# Supplementary material for: Rationales and functions of disliked music: An in-depth interview study
Source: PLoS One. 2022 Feb 15;17(2):e0263384. doi: 10.1371/journal.pone.0263384 (PMC8846515; doi:10.1371/journal.pone.0263384)
Supplement: S5 Table — (PDF) [file pone.0263384.s007.pdf]

Table S5

Sum of Frequencies of Reference Points of Disliked Music

**Ref. Point**	***N***	**% (von total *N* = 277)**
**M***	235	84,84%
**L***	91	32,85%
**A***	87	31,41%
**F***	56	20,22%
**P***	44	15,88%

Note. Sum of all combinations per reference point. For example, M* means all combinations of Music with the other reference points such as Lyrics, Artist, Fan/Listener, and Performer.
