# Supplementary material for: Rationales and functions of disliked music: An in-depth interview study
Source: PLoS One. 2022 Feb 15;17(2):e0263384. doi: 10.1371/journal.pone.0263384 (PMC8846515; doi:10.1371/journal.pone.0263384)
Supplement: S1 File — (PDF) [file pone.0263384.s008.pdf]

Supporting Information

Rationales and Functions of Disliked Music: An In-Depth Interview Study

Taren-Ida Ackermann & Julia Merrill

Methods

In preparation for the interview ask participants to think in advance about what music they do not like (bring in a list, and if possible, bring exemplary audio files).

Interview Guide

- What music do you dislike? Are there any music, performers, groups, or styles that you dislike?
- What music do you not like at all / dislike? Pieces, artists, styles?
- For what reason do you reject this music in each case?
- What do you associate with the disliked music?
- Do you talk about your dislikes with others?
- Rate each dislike on a scale of 1-10.
- What do you do when this music is played somewhere?

In general

- What characteristics does music have that you dislike?
- Are there general descriptions for music you dislike?

Disliked music in the process

- How did your dislikes come about?
- Have your dislikes changed over time?
- Is there music you used to dislike and now like?
- Is there music you used to like and now dislike?
- How did these changes come about? What has changed?

Influencing factors

- What does it depend on whether you like or dislike music? Are there situational influencing factors?
- Does the judgment of others influence your opinion about disliked music?
- Does your environment share your musical dislikes?

Toward the end

- What does your disliked music say about you?
